# Ultrasonic-Assisted Heterogeneous Fenton-like (UHEF) Pretreatment of Eucalyptus Sawdust for Enhanced Enzymatic Hydrolysis

**DOI:** 10.3390/molecules31162926

**Published:** 2026-08-21

**Authors:** Han Zhang, Cuixian Peng, Xiaoguo Wang, Ping Li, Wei Tan, Shujie Wang

**Affiliations:** 1 School of Agronomy, Chuxiong Normal College, Chuxiong 675000, China; 2 Wenshan Academy of Agricultural Sciences, Wenshan 663000, China

**Keywords:** ultrasonic, Fenton-like system, pretreatment, eucalyptus sawdust, enzymatic hydrolysis

## Abstract

Ultrasonic-assisted heterogeneous Fenton-like (UHEF) pretreatment is an emerging strategy that overcomes the limitations of traditional homogeneous Fenton systems while enhancing the efficiency of lignocellulosic biomass processing. In this study, we constructed a UHEF system employing iron-loaded zeolite as a heterogeneous catalyst to pretreat eucalyptus sawdust (ES). Process conditions were optimized using response surface methodology (RSM) with a central composite design (CCD), yielding a maximum reducing sugar production of 441.45 mg/g. We systematically investigated the mechanism by which UHEF pretreatment enhances the enzymatic hydrolysis of ES through compositional analysis, Fourier transform infrared (FTIR) spectroscopy, X-ray diffraction (XRD), cross-polarization magic-angle spinning carbon-13 nuclear magnetic resonance (CP/MAS ^13^C NMR), and scanning electron microscopy (SEM). Additionally, recovery and recycling experiments were conducted to evaluate the reusability of the iron-loaded zeolite. XRD, XPS, and ICP-OES were further used to verify the crystalline structure, surface Fe chemical states, and Fe loading of the catalyst, and Fe leaching was quantified for each reuse cycle. The results demonstrated that UHEF pretreatment effectively removed lignin and hemicellulose from ES, disrupted the cellulose crystalline structure, and generated numerous grooves on the substrate surface. These modifications increased the effective adsorption of cellulase and enhanced reducing sugar production to 4.32 times that of raw eucalyptus sawdust (RES). Although the recycling experiments indicated that the stability of the iron-loaded zeolite requires further improvement, the catalyst retained a certain degree of reusability over four consecutive cycles. These findings demonstrate that UHEF pretreatment is a promising approach with broad application prospects in lignocellulosic biorefinery, consistent with recent advances in advanced oxidation processes for biomass valorization.

## 1. Introduction

The escalating consumption of fossil resources has precipitated a global energy crisis and severe environmental degradation, including the greenhouse effect driven by CO_2_ emissions [1,2]. In response, the development of sustainable and environmentally benign strategies for advanced biofuel production has garnered considerable attention worldwide. Lignocellulosic biomass represents a particularly promising feedstock for biofuel production owing to its abundance, renewability, and high cellulose content, which can be hydrolyzed to monomeric sugars and subsequently fermented to ethanol and other value-added products [3,4]. However, the rigid cellulose–hemicellulose–lignin network and the inherent crystallinity of cellulose present formidable barriers to enzymatic hydrolysis, necessitating an effective pretreatment step to disrupt these three-dimensional structural and crystalline obstacles, thereby enhancing enzyme accessibility to cellulose.

Numerous pretreatment methodologies have been investigated for lignocellulosic biomass, including mechanical, thermal, acid, alkaline, and oxidative approaches, as well as various combinations thereof [1]. Among these, advanced oxidation processes (AOPs) have emerged as particularly effective for biomass-to-biofuel conversion. Chan and Epelle comprehensively reviewed AOP-based biomass pretreatment and reported that a combined Fenton-ultrasound-alkali system achieved 74% lignin reduction and a 106.45% increase in cellulose content for sugarcane bagasse [2]. Zhou provided an extensive review of oxidative pretreatment methods for enzymatic hydrolysis, concluding that Fenton oxidation combined with other pretreatment strategies produces synergistic effects that overcome the recalcitrance of lignocellulose for highly efficient fermentable sugar release [3]. These findings underscore the substantial potential of hybrid oxidation approaches for lignocellulosic biomass valorization.

Fenton oxidation, first reported by Fenton in 1894 [4], has emerged as a low-cost and effective approach for degrading lignocellulose under ambient temperature and pressure conditions. In a Fenton system, iron acts as a catalyst to decompose hydrogen peroxide into hydroxyl radicals, which subsequently react with lignocellulosic components [5]. Specifically, Fe^2+^ catalyzes the decomposition of H_2_O_2_ to generate hydroxyl radicals (HO·) at pH 3–5, while the resulting Fe^3+^ reacts further with H_2_O_2_ to regenerate Fe^2+^. The formation of reactive oxygen species (ROS), including hydroxyl radicals (HO·) and hydroperoxyl radicals (HOO·), initiates the nonselective oxidation of lignocellulosic components. Sheng recently demonstrated that the Fenton-assisted hydrothermal humification of corn stalk achieved a 38.55% increase in fulvic acid yield through a depolymerization–oxidation–reconstruction pathway, highlighting the efficacy of hydroxyl radical-mediated biomass deconstruction [6]. When miscanthus, switchgrass, corn stover, and wheat straw were treated with Fenton reagent, an average increase of 212% in enzymatic saccharification was achieved [7]. Rajendran and Vaidyanathan further demonstrated that a bio-Fenton oxidation system achieved 595 mg/g fermentable sugar from Casuarina equisetifolia biomass at the pilot scale, providing compelling evidence for the scalability of Fenton-based biomass pretreatment [8].

Despite these advantages, conventional homogeneous Fenton oxidation suffers from several inherent limitations that constrain its practical application in biomass processing. As solution pH increases, various Fe^3+^ complexes form, leading to the generation of substantial quantities of ferric hydroxide sludge that not only reduces pretreatment efficiency, but also creates significant disposal challenges [9,10,11]. To mitigate iron precipitation, large quantities of acid must be added to maintain the reaction medium at pH 3–5 [12]. However, these acidic conditions may promote the formation of inhibitory compounds, including acetic acid, 5-hydroxymethylfurfural (HMF), and furfural, which are toxic to subsequent enzymatic hydrolysis and fermentation [13]. Additionally, the homogeneous catalyst cannot be recovered and reused, increasing both operational costs and environmental impact.

Heterogeneous Fenton-like catalysis has emerged as a promising strategy to overcome these limitations. By immobilizing iron species on solid supports such as zeolites, biomass-derived carbon, metal-organic frameworks (MOFs), and porous carbon frameworks, heterogeneous catalysts enable sustained H_2_O_2_ decomposition over a wider pH range while eliminating sludge formation [5,14]. Zhang developed biomass-derived iron-doped porous carbon that achieved 94.4% tetracycline removal within 15 min across a broad pH range of 3–9, demonstrating the superior pH tolerance of heterogeneous systems [14]. Tang synthesized Mn-doped Fe-gallate MOFs on biomass cotton fibers that achieved 90.3% tetracycline removal with effective operation from pH 2 to 9, highlighting the potential of bimetallic synergy on biomass supports [15]. Yang fabricated CoFe_2_O_4_ spinel on biomass-derived 3D porous carbon, achieving 97.5% ciprofloxacin removal through peroxymonosulfate (PMS) activation with efficient Co^2+^/Co^3+^ and Fe^2+^/Fe^3+^ redox cycling [16]. These studies collectively demonstrate that heterogeneous catalysts not only prevent iron hydroxide precipitation, but also maintain the ability to generate hydroxyl radicals from H_2_O_2_ while enabling facile recovery and recycling [9,10].

Iron-loaded zeolites are particularly attractive heterogeneous Fenton catalysts due to the unique properties of zeolitic materials. Zeolites are hydrated aluminosilicates with three-dimensional cage-like frameworks that provide specific surface areas of several hundred square meters per gram and cation exchange capacities of up to several milliequivalents per gram [17]. The strong adsorption capacity of zeolites enables the firm binding of iron cations to exchange sites, minimizing leaching during reaction. Pan demonstrated that FeOCl supported on biomass-derived activated carbon achieved greater than 91% dichloroethane removal with sustained Fe^2+^/Fe^3+^ cycling on the catalyst surface [5], while Ye reported that biomass-derived carbon-coated ferrous sulfide achieved 94.08% phenol removal through synergistic radical and non-radical pathways [18]. Li reviewed recent advances in electrochemical applications of biochar and confirmed that Fe-doped biochar has become the most sought-after Fenton electrode material, with Fe_3_O_4_-loaded biochar achieving 95% chemical oxygen demand (COD) removal [7]. These findings support the use of iron-supported materials as effective heterogeneous Fenton catalysts for biomass processing.

The integration of ultrasound with heterogeneous Fenton-like systems offers additional synergistic benefits for biomass pretreatment. Ultrasound irradiation generates acoustic cavitation, producing localized temperatures of approximately 5000 K and pressures of 1000 atm upon bubble collapse [19]. These extreme conditions facilitate the homolytic cleavage of water molecules to generate additional hydroxyl radicals (HO·) and hydrogen atoms (H·), thereby augmenting the radical concentration in the reaction system [19]. Furthermore, ultrasound enhances mass transfer, cleans catalyst surfaces in real-time, increases free radical production, and removes resistance to mass transfer from the liquid phase to the catalyst surface [19]. Chai demonstrated that ultrasound-assisted Fenton pretreatment using acid mine drainage as a natural iron catalyst achieved 13.51 wt% 5-hydroxymethylfurfural (HMF) yield from raw biomass, with the synergy between ultrasound and Fenton oxidation effectively destroying the lignocellulose structure [9]. Chan and Epelle further reported that multistage combinations of Fenton, ultrasound, and alkali consistently outperformed dual combinations in delignification, cellulose crystallinity reduction, and microbial compatibility [2].

While previous studies have demonstrated the effectiveness of heterogeneous Fenton-like catalysts for environmental remediation and the potential of ultrasound-Fenton combinations for biomass processing, several critical knowledge gaps remain. Systematic optimization of ultrasound-assisted heterogeneous Fenton-like (UHEF) pretreatment using statistical approaches such as response surface methodology (RSM) has been limited. Furthermore, comprehensive mechanistic characterization of the effects of UHEF pretreatment on lignocellulosic biomass structure, including detailed analysis of cellulose crystallinity changes, surface morphology modifications, and catalyst recyclability, remains insufficiently explored.

In this study, we developed an ultrasound-assisted heterogeneous Fenton (UHEF) system employing Fe^2+^-loaded zeolite combined with hydrogen peroxide to pretreat eucalyptus sawdust (ES). The operational parameters of the UHEF pretreatment were optimized using RSM to maximize sugar yield. We comprehensively compared the effects of UHEF pretreatment with those of an ultrasound-assisted homogeneous Fenton (UHOF) pretreatment to evaluate their respective impacts on the composition and structural characteristics of eucalyptus biomass. Furthermore, we assessed the recyclability of the Fe^2+^-loaded zeolite catalyst for repeated ES pretreatment cycles. Given that heterogeneous Fenton systems can process eucalyptus biomass in a green, sustainable, and efficient manner, we anticipate that this approach holds substantial potential for application in lignocellulosic biorefinery. This work builds on our previous two-stage NaOH/[Bmim]Cl pretreatment of waste medium-density fiberboard [20] and extends the pretreatment concept to an ultrasound-assisted heterogeneous Fenton-like system.

## 2. Results and Discussion

### 2.1. Preparation of Fe-Exchanged Zeolite

Natural zeolites typically exhibit relatively low ion exchange capacity. Therefore, we initially treated the zeolite with NaOH under microwave irradiation, as the ion exchange capacity of zeolites tends to increase with the relative content of low-valence, large-radius alkali metal cations [21,22].

The ferrous ion loading capacity was quantitatively evaluated by measuring the residual iron content in the solution after the adsorption process. The optimal Fe^2+^ loading was investigated by varying the zeolite-to-ferrous sulfate ratio from 1:0.2 to 1:4 (*w*/*w*). The Fe^2+^ adsorption efficiency is presented in Figure S1. As expected, the modified zeolite exhibited strong ferrous ion adsorption capacity, with adsorption efficiencies exceeding 90% across all tested conditions. Specifically, when the zeolite mass was fixed at 1 g, the Fe^2+^ adsorption efficiency increased from 93.5% to 96.2% as the FeSO_4_ dosage increased from 0.2 g to 1 g. However, the adsorption efficiency remained essentially unchanged when the ferrous sulfate dosage was further increased from 1 g to 4 g, suggesting that the adsorption capacity of the modified zeolite approached saturation at FeSO_4_ dosages exceeding 1 g. Consequently, all subsequent experiments were conducted using a zeolite-to-ferrous sulfate ratio of 1:1 (*w*/*w*) for the Fe-exchanged zeolite preparation.

The Fe^2+^ adsorption efficiency observed in this study (>93%) is comparable to that reported for other biomass-based iron immobilization systems. Lu et al. developed an Fe^2+^-sodium alginate-biomass carbon hydrogel that achieved effective iron chelation for phenol degradation [10], while Tang et al. reported that Mn-doped Fe-gallate MOFs on biomass cotton fibers exhibited strong metal binding through coordination with gallic acid ligands [15]. The high adsorption efficiency of our NaOH-modified zeolite confirms its suitability as a robust support for heterogeneous Fenton catalysis in biomass pretreatment applications.

**Figure 1:**
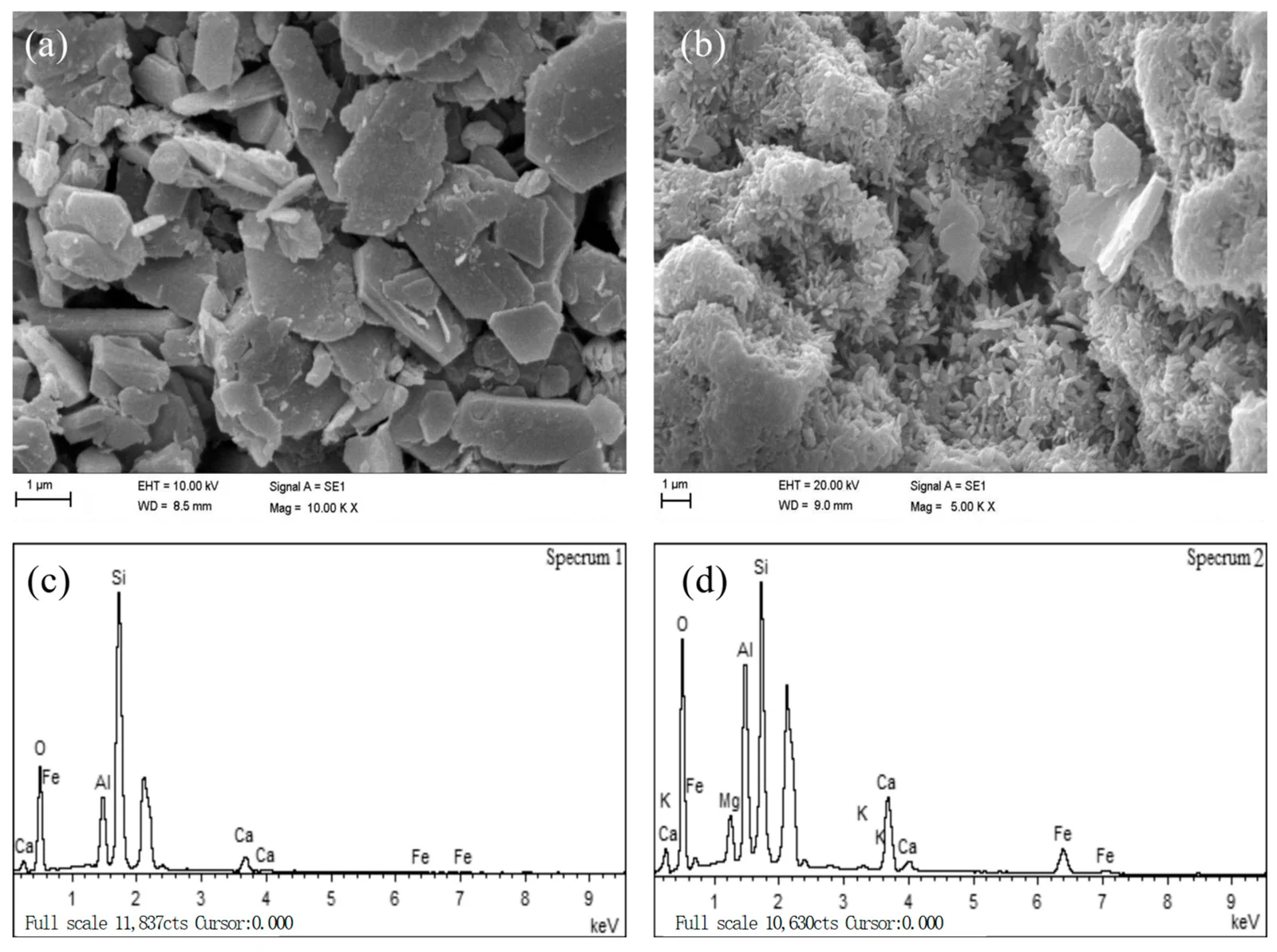
SEM images and SEM-EDX spectra. (**a**) Raw modified zeolite, (**b**) iron coated modified zeolite, (**c**) EDX of raw modified zeolite, and (**d**) EDX of iron coated modified zeolite.

Figure 1a,b shows the surface of raw modified zeolite and iron coated modified zeolite, respectively. As shown in Figure 1b, the surface of the iron coated modified zeolite displayed rod-shaped iron oxide particles, which did not appear on the surface of the raw modified zeolite. The SEM-EDX spectra shown in Figure 1c,d supported the same conclusion. The SEM-EDX spectrum of iron coated modified zeolite showed a strong peak of iron, whereas no iron peak was detected in the spectrum of the raw modified zeolite, further confirming that iron was successfully immobilized on the modified zeolite.

#### XRD, XPS, and ICP-OES Characterization of the Catalysts

**Figure 2:**
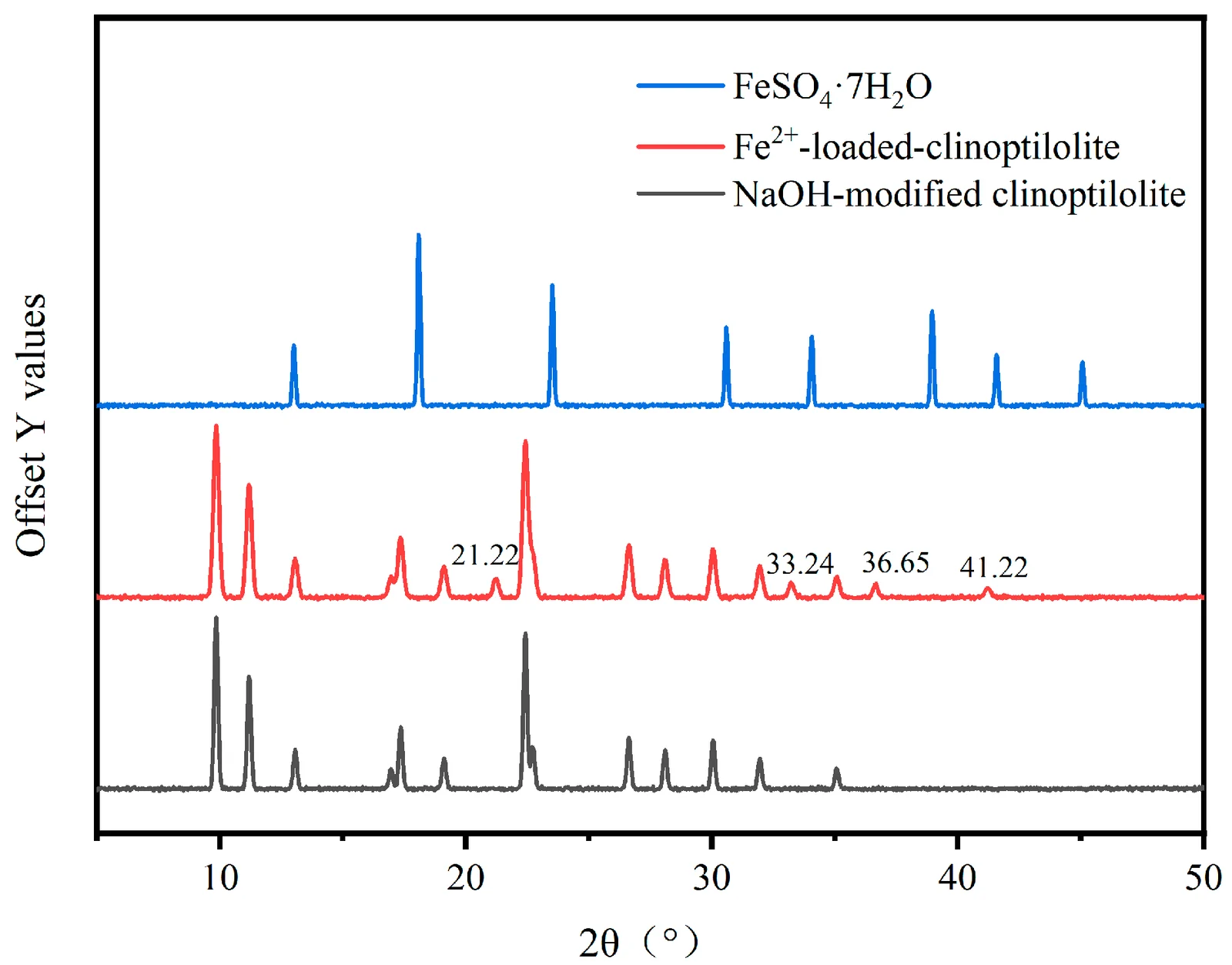
XRD patterns of NaOH-modified clinoptilolite, Fe^2+^-loaded clinoptilolite, and FeSO_4_·7H_2_O.

The crystalline structures of the NaOH-modified clinoptilolite, Fe^2+^-loaded clinoptilolite, and FeSO_4_·7H_2_O were compared by XRD (Figure 2). The modified zeolite retained the characteristic clinoptilolite reflections at 2θ = 9.85°, 11.19°, 22.42°, and 26.63°. After Fe^2+^ loading, these reflections remained at essentially unchanged positions but decreased in intensity and broadened slightly, indicating successful iron immobilization without collapse of the zeolite framework. Additional weak reflections at 21.22°, 33.24°, 36.65°, and 41.22° are consistent with trace goethite-like FeOOH formed by partial surface hydrolysis/oxidation, in agreement with the rod-like iron oxide particles observed by SEM. No intense FeSO_4_·7H_2_O reflections were detected in the loaded catalyst, indicating that the iron phase is highly dispersed rather than present as crystalline FeSO_4_.

**Figure 3:**
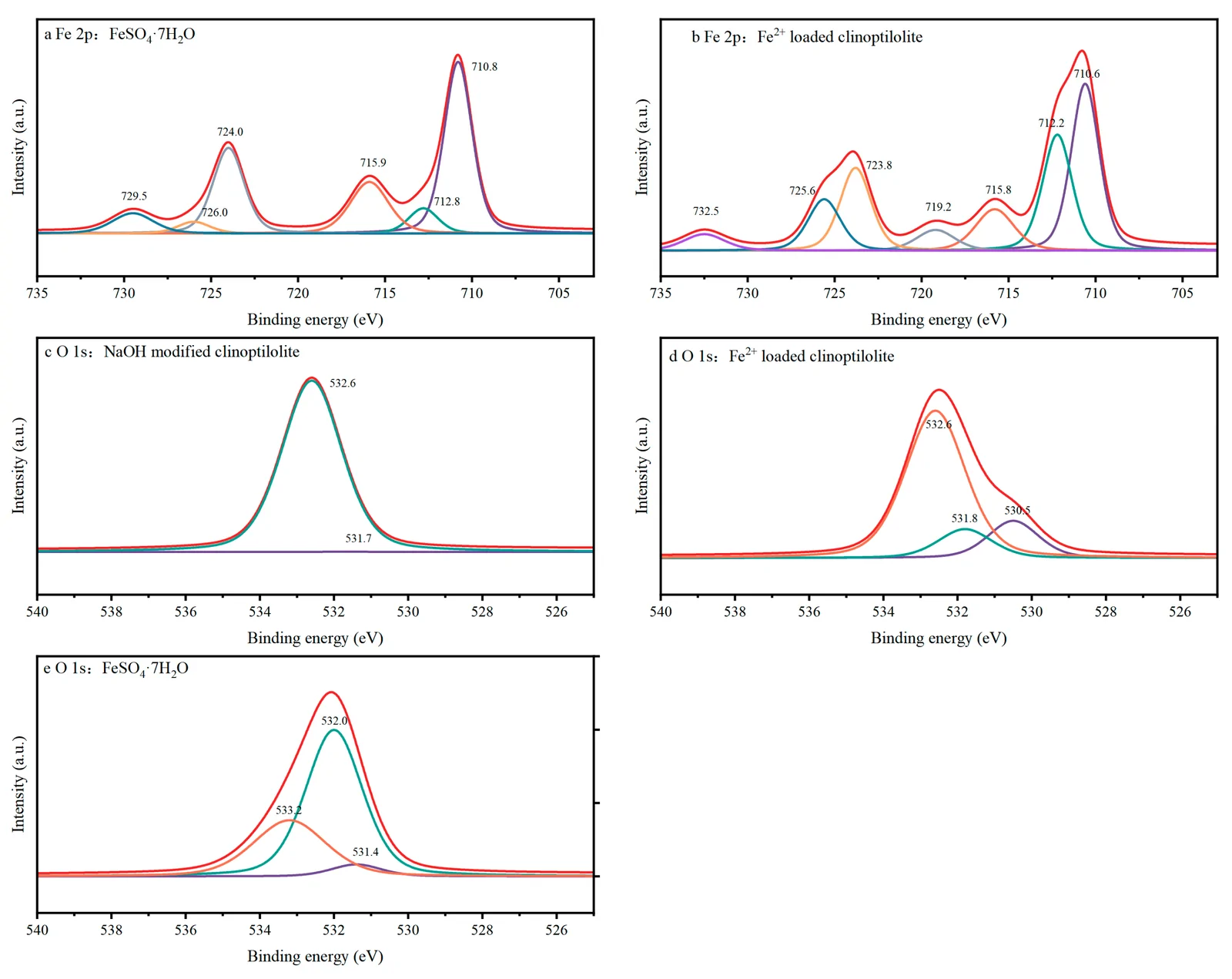
XPS spectra of NaOH-modified clinoptilolite, Fe^2+^-loaded clinoptilolite, and FeSO_4_·7H_2_O.

XPS was used to verify the surface chemical states of iron (Figure 3). The survey spectrum of the Fe^2+^-loaded zeolite showed new Fe 2p, Fe 3p, and weak S 2p signals relative to the NaOH-modified zeolite. In the Fe 2p region, the loaded catalyst exhibited Fe^2+^ 2p3/2 at 710.6 eV with a satellite at 715.8 eV, together with Fe^3+^ components at 712.2 and 725.6 eV, indicating that exchanged Fe^2+^ coexists with partially oxidized surface Fe^3+^. The O 1s spectrum of the loaded catalyst contained an additional Fe–O component at 530.5 eV and a surface –OH component at 531.8 eV in addition to the framework Si–O–Al oxygen at 532.6 eV. These features support iron–oxygen coordination at the zeolite surface.

**Figure 4:**
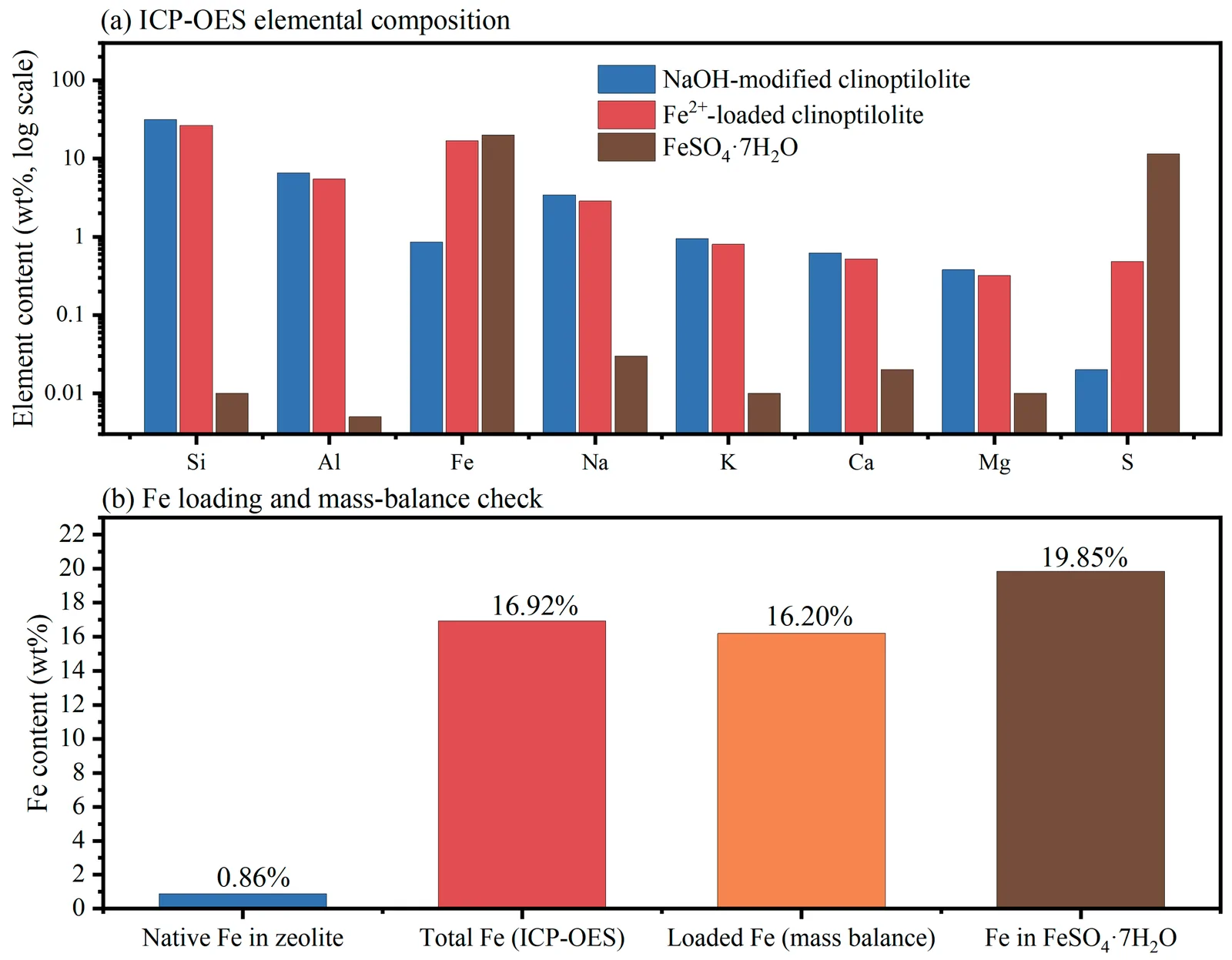
ICP-OES elemental composition and Fe loading mass-balance check for the catalysts.

ICP-OES analysis confirmed the iron loading (Table 1 and Figure 4). The Fe^2+^-loaded catalyst contained 16.92 wt% Fe. This value agrees with the mass-balance estimate of 16.20 wt% loaded Fe calculated from the 1:1 (*w*/*w*) zeolite/FeSO_4_·7H_2_O ratio, the Fe content of FeSO_4_·7H_2_O (20.09%), and the measured Fe^2+^ adsorption efficiency (96.2%). A small S signal (0.48 wt%) was attributed to residual sulfate after washing.

### 2.2. Optimization of Sugar Yield for UHEF Pretreated Eucalyptus

The UHEF pretreatment process was optimized using RSM with CCD to maximize the reducing sugar yield. The individual and interactive effects of H_2_O_2_ concentration (*A*), ultrasound power (*B*), and pretreatment time (*C*) on the reducing sugar yield were systematically investigated (Table S2). Regression analysis of the experimental data yielded the following quadratic equation (Equation (1)), where *Y* represents the reducing sugar yield:

(1) $$\begin{array}{l} Y=418.78+1.96\times A+4.90\times B+24.40\times C\\ -14.96\times A\times B+6.07\times A\times C-8.57\times B\times C\\ -16.68\times A^{2}-8.96\times B^{2}-15.39\times C^{2} \end{array}$$

The quadratic model was selected as it provided the best fit to the experimental results.

The ANOVA results are summarized in Table 2. The model exhibited a high coefficient of determination (R^2^ = 0.9471), indicating that 94.71% of the variability in the response was explained by the model. The adjusted coefficient of determination (adjusted R^2^ = 0.8995) further confirmed the high significance of the model. The F-test for ANOVA yielded a model F-value of 19.90, indicating that the model was statistically significant (*p* < 0.0001). There was only a 0.01% probability that this F-value could have occurred due to random noise. Model terms with *p*-values less than 0.0500 were considered statistically significant. In this study, the *p*-values for H_2_O_2_ concentration (*A*, *p* < 0.05) and pretreatment time (*C*, *p* < 0.05) were both significant; in addition, the AB interaction and the quadratic terms A_2_, B_2_, and C_2_ were also significant, whereas the linear effect of ultrasound power (*B*) was not statistically significant. These results indicate that H_2_O_2_ concentration and pretreatment time were the primary factors influencing the reducing sugar yield. The non-significant lack-of-fit (*p* = 0.4485) further confirmed that the model adequately described the experimental data.

The three-dimensional response surface plots (Figure S2) illustrate the interactions between the experimental factors, with each plot displaying the effect of two factors while the third was maintained at its center point (zero level). The optimal conditions were determined based on the model predictions: H_2_O_2_ concentration of 3.003%, ultrasound power of 267.965 W, and pretreatment time of 74.458 min. Under these optimized conditions, the model predicted a maximum reducing sugar yield of 442.415 mg/g.

To validate the reproducibility and accuracy of the model, three replicate pretreatment experiments were conducted under the optimized conditions. For practical operability, the parameters were adjusted to an H_2_O_2_ concentration of 3%, ultrasound power of 270 W, and pretreatment time of 74 min. The average reducing sugar yield of the three replicates was 441.45 ± 6.87 mg/g, which was in excellent agreement with the predicted value. This result confirms the validity and reliability of the response surface model.

To isolate the contributions of ultrasound, H_2_O_2_, and the heterogeneous catalyst, control experiments were conducted under otherwise identical conditions (Table S3 and Figure S3a). Ultrasound alone (118.35 ± 5.02 mg/g) and H_2_O_2_ alone (132.48 ± 6.15 mg/g) produced only marginal increases over RES (102.03 ± 4.21 mg/g). The homogeneous Fenton system without ultrasound (HOF, 289.64 ± 8.33 mg/g) and the heterogeneous system without ultrasound (HEF, 351.27 ± 9.48 mg/g) were both less effective than their ultrasound-assisted counterparts (UHOF, 326.32 ± 7.92 mg/g; UHEF, 441.45 ± 6.87 mg/g). The higher yield of UHEF relative to UHOF and HEF demonstrates the synergistic contribution of acoustic cavitation and heterogeneous Fe^2+^/H_2_O_2_ activation.

The reducing sugar yield of 441.45 mg/g achieved in this study compares favorably with values reported in the recent literature for Fenton-based biomass pretreatment. Rajendran and Vaidyanathan [8] achieved 595 mg/g fermentable sugar from Casuarina equisetifolia biomass using a bio-Fenton oxidation system at pilot scale (25 L bioreactor), though their starting biomass contained a higher cellulose content (55.69%) compared to our eucalyptus sawdust (41.35%). Chan and Epelle [2] reported that Fenton combined with ultrasound and alkali achieved substantial delignification (74%) and cellulose content increase (106.45%) for sugarcane bagasse. Karthikeyan et al. [11] demonstrated that integrated Fenton and plasma pretreatment achieved 26% biomethane yield increase from maize biomass with 32% delignification. The sugar yield obtained in our study represents a 4.32-fold increase over raw eucalyptus sawdust, demonstrating the effectiveness of the UHEF system for enhancing the enzymatic hydrolysis of recalcitrant hardwood biomass.

### 2.3. Composition Analysis

**Figure 5:**
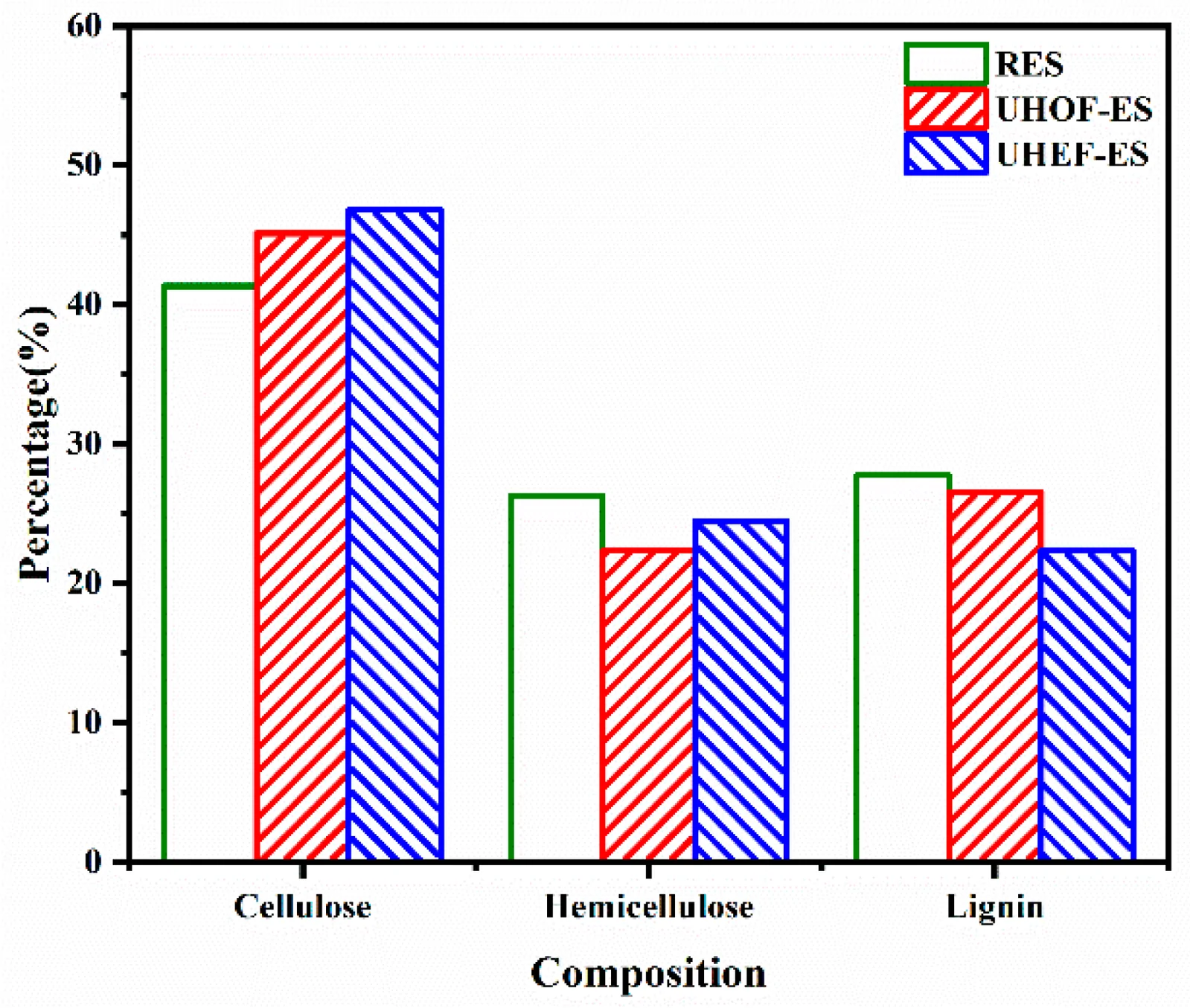
Changes of ES components before and after pretreatment.

Pretreatment typically induces changes in the relative proportions of the principal lignocellulosic components. The removal of non-cellulosic components, particularly hemicellulose and lignin, which impede enzymatic hydrolysis, is beneficial for enhancing cellulose digestibility and increasing fermentable sugar production [23]. Therefore, we performed a compositional analysis of native (RES), UHOF-pretreated (UHOF-ES), and UHEF-pretreated (UHEF-ES) eucalyptus sawdust, and the results are presented in Figure 5.

RES contained 41.35% cellulose, 26.31% hemicellulose, and 27.79% Klason lignin on a dry weight basis. Following UHOF pretreatment, the hemicellulose content decreased substantially from 26.31% to 22.34%, representing a greater relative change than that observed for UHEF-pretreated samples. This result suggests that the UHOF pretreatment was more effective at hemicellulose removal. In contrast, the UHEF pretreatment achieved a more pronounced reduction in lignin content, with a delignification efficiency of approximately 19.5%, demonstrating its superior capacity for lignin removal. The substantial removal of both hemicellulose and lignin resulted in a significant increase in the relative cellulose content of the pretreated ES, indicating that a greater proportion of cellulose would be available to participate in the hydrolysis reaction, thereby increasing reducing sugar production [22]. Comparing the compositional changes induced by the two pretreatment methods, the UHEF-pretreated ES exhibited a higher relative cellulose content, suggesting that UHEF pretreatment would yield greater enzymatic hydrolysis efficiency [22].

These compositional changes are consistent with recent reports on Fenton-based biomass pretreatment. Sheng et al. [6] reported that Fenton-assisted hydrothermal treatment of corn stalk resulted in 89.01% hemicellulose conversion while retaining 81.89% of cellulose, demonstrating the selective fractionation capability of Fenton oxidation. Yang et al. [12] showed that Fenton-like pretreatment of biomass effectively deconstructed lignocellulosic macromolecules, doubling the specific surface area to 2801 m^2^/g through hydroxyl radical attack on cellulose, hemicellulose, and lignin. Chan and Epelle [2] reported that Fenton-ultrasound-alkali combinations reduced hemicellulose by 48.34% for sugarcane bagasse. The preferential delignification observed in our UHEF system aligns with the established mechanism whereby hydroxyl radicals generated in Fenton systems preferentially attack lignin structures due to their higher reactivity toward aromatic compounds [24].

### 2.4. FTIR Analysis

**Figure 6:**
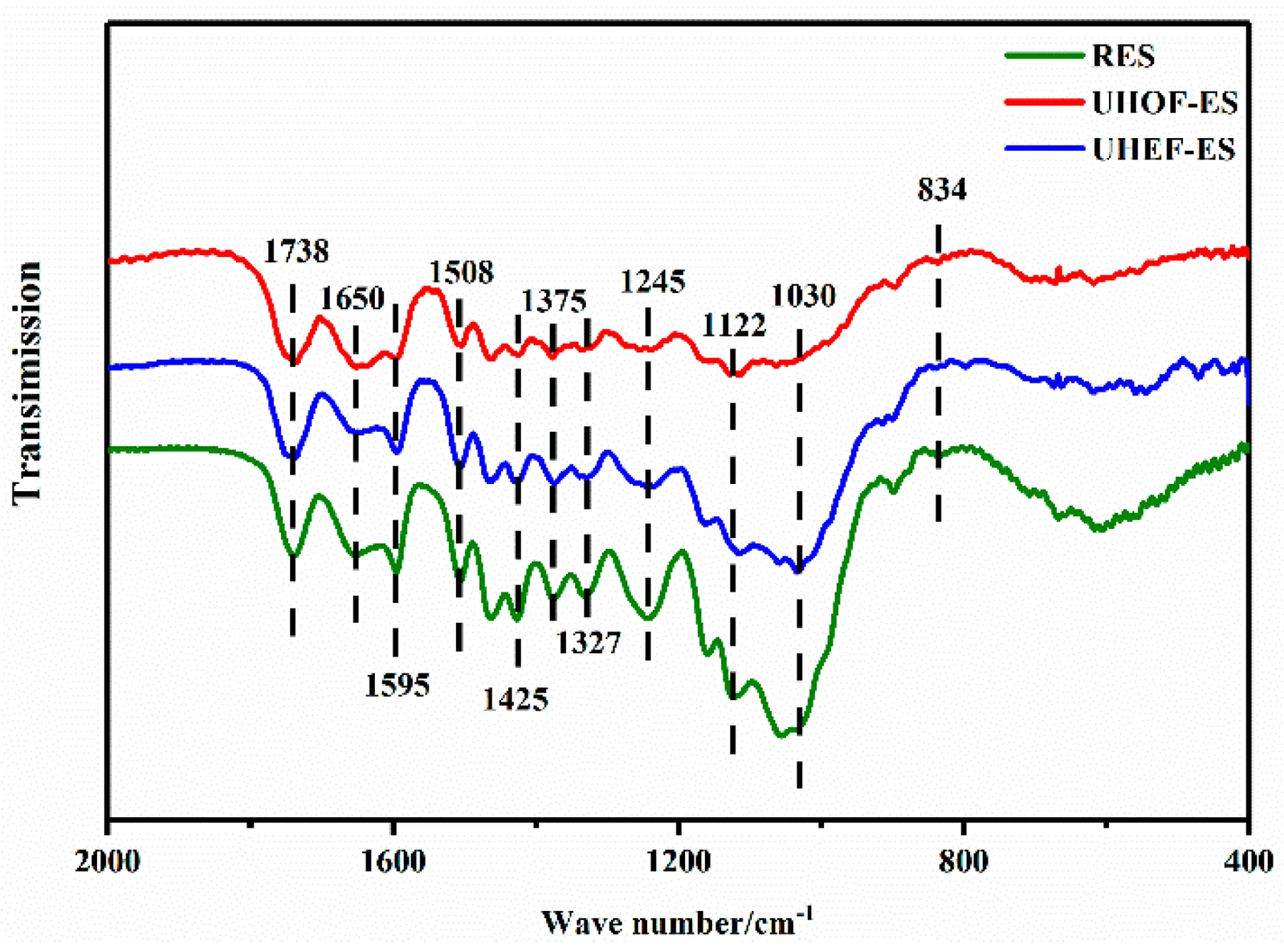
ES infrared spectra before and after pretreatment.

The FTIR spectra of all ES samples are presented in Figure 6. The absorption band at 1738 cm^−1^ is attributed to the carbonyl stretching vibration of acetyl and uronic ester groups in hemicellulose [25]. Compared with the spectrum of RES, the absorption peak at this position for UHOF-ES exhibited increased peak broadening and reduced intensity, indicating the cleavage of ester linkages in hemicellulose during pretreatment. Similarly, the absorption bands at 1375 cm^−1^ and 1030 cm^−1^, corresponding to the C-H bending and C-O stretching vibrations of hemicellulose, respectively [25,26], showed significant intensity reductions following UHOF pretreatment. These spectral changes confirm the degradation and partial removal of hemicellulose from ES during UHOF pretreatment.

Additionally, the characteristic absorption bands associated with the aromatic skeleton vibrations of lignin at 1650 cm^−1^, 1595 cm^−1^, 1508 cm^−1^, and 1425 cm^−1^ [25,26] exhibited significant intensity decreases following UHOF pretreatment, indicating substantial disruption of the lignin aromatic ring structure. In contrast, the UHEF-ES spectrum showed that these phenomena were less pronounced for hemicellulose-related bands. However, the absorption bands characteristic of syringyl (S) units at 1327 cm^−1^ and 1122 cm^−1^ and guaiacyl (G) units at 1245 cm^−1^ and 834 cm^−1^ [26,27] exhibited more pronounced peak broadening and intensity reduction compared with UHOF-ES. These observations indicate a more extensive removal of both syringyl and guaiacyl lignin structural units during UHEF pretreatment, which is consistent with the compositional analysis results showing lower residual lignin content in UHEF-ES.

The selective attack on lignin aromatic structures observed in our FTIR analysis is consistent with molecular-level studies of the hydroxyl radical oxidation of biomass. Fan et al. investigated the molecular-level transformations of biomass burning-derived water-soluble organic carbon during dark aqueous OH oxidation and found that polyphenols were the most susceptible compounds to degradation, while CHO compounds experienced substantial oxidation with O/C ratios increasing from 0.35 to 0.48 [24]. These findings support the preferential degradation of lignin aromatic units observed in our UHEF-ES samples. Yang et al. similarly reported that hydroxyl radicals from Fenton-like reagents oxidized lignin, cellulose, and hemicellulose in biomass, destroying the network structure of macromolecules and increasing porosity [12]. Zhou et al. noted that Fenton oxidation modifies lignin surfaces through the oxidation of phenolic hydroxyl groups and an increase in carboxylic groups, which reduces the non-productive adsorption of cellulases on lignin, thereby enhancing enzymatic hydrolysis efficiency [3].

### 2.5. XRD Analysis

XRD analysis was performed on all ES samples to investigate the effects of UHEF pretreatment on the crystalline structure and crystallinity index (CrI), thereby providing insight into the relationship between structural changes and enzymatic hydrolysis efficiency.

**Figure 7:**
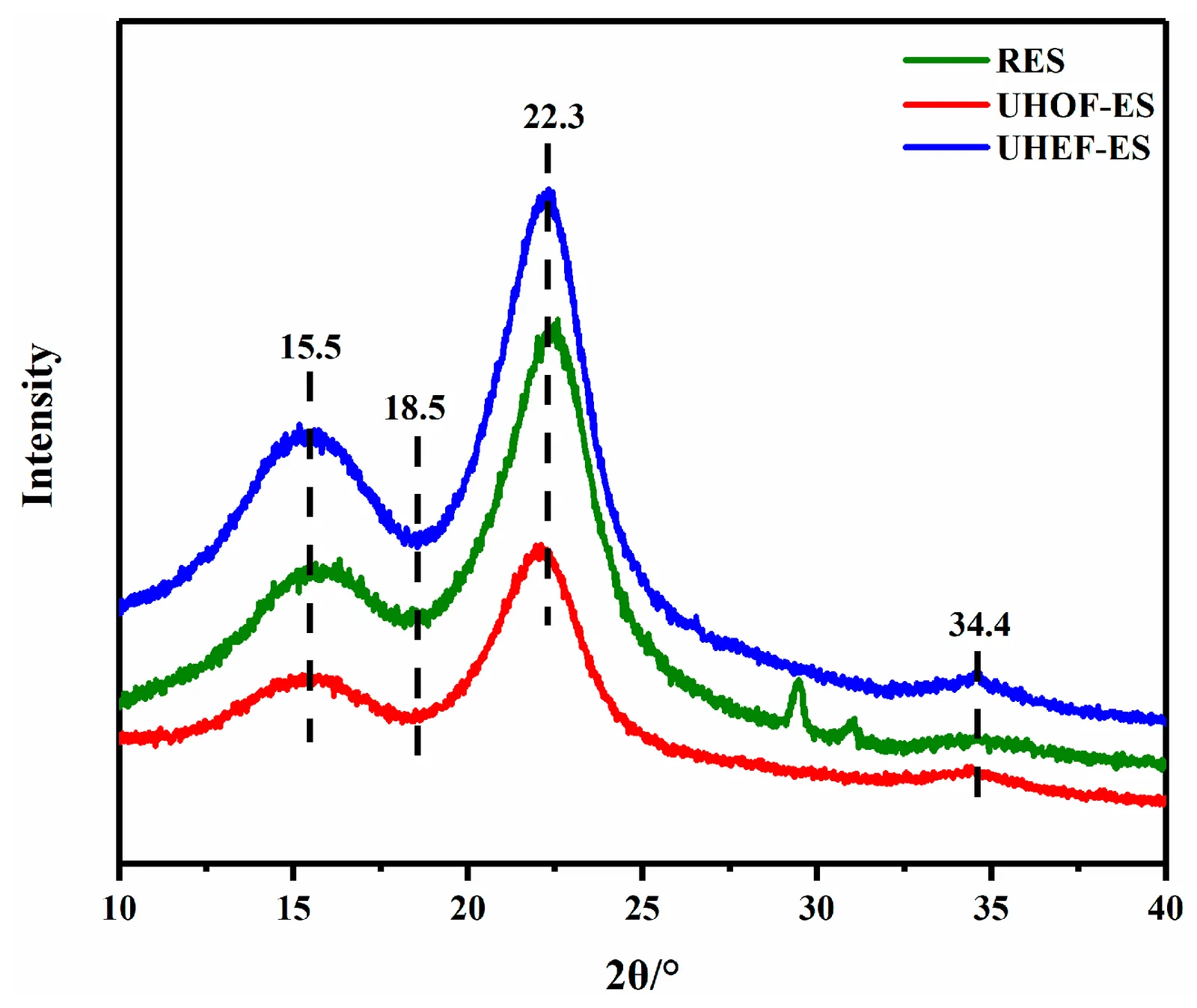
ES XRD patterns before and after pretreatment.

As shown in Figure 7, all XRD patterns exhibited characteristic diffraction peaks corresponding to the (110), (002), and (040) crystallographic planes at 2θ values of approximately 15.5°, 22.3°, and 34.3°, respectively, confirming that the cellulose I allomorph structure was preserved after both pretreatments; only the diffraction peak intensities were altered [27]. Specifically, compared with RES, the UHOF-ES pattern showed broadening and flattening of the peak at 15.5°, indicating partial disruption of the crystalline structure and a concomitant decrease in the crystallinity index. To quantify these changes, the CrI of each sample was calculated using the Segal method (Table S4). The CrI of UHOF-ES decreased from 53.80% to 52.67%, which is expected to enhance cellulase accessibility and improve enzymatic hydrolysis performance [28].

In contrast, the XRD pattern of UHEF-ES exhibited sharper and more intense diffraction peaks for all crystallographic planes, suggesting an apparent increase in crystallinity. The calculated CrI of UHEF-ES was higher than that of RES (Table S4). This increase in CrI is attributed to the preferential removal of amorphous components (hemicellulose and lignin) during pretreatment, which results in a relative enrichment of crystalline cellulose in the residual biomass [28]. Previous studies have demonstrated that the removal of amorphous components reduces nonproductive cellulase binding and enhances the effective action of cellulase on cellulose, ultimately increasing reducing sugar production [27]. Chan and Epelle [2] similarly reported that multistage AOP combinations including Fenton and ultrasound consistently reduce cellulose crystallinity while improving delignification and microbial compatibility for subsequent bioprocessing.

### 2.6. ^13^C CP/MAS Solid State NMR

**Figure 8:**
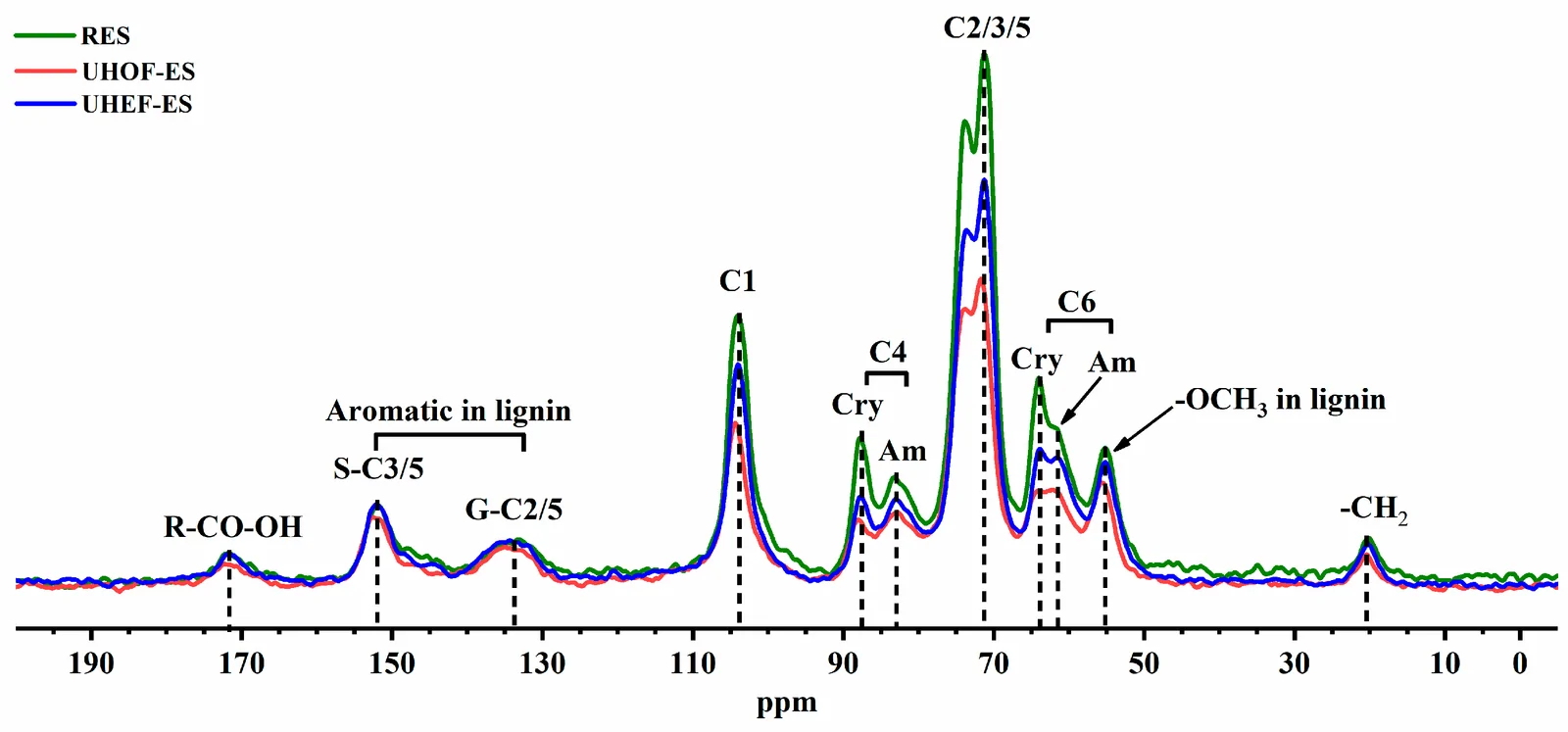
^13^C NMR spectra before and after pretreatment.

The CP/MAS ^13^C NMR spectra of RES, UHOF-ES, and UHEF-ES are presented in Figure 8.The signals observed at 21 ppm and 171 ppm in the RES spectrum are assigned to the methyl carbon and carboxyl/carbonyl carbon of hemicellulose acetyl groups, respectively [22]. Following UHOF pretreatment, the intensities of these signals were substantially attenuated, indicating a reduction in hemicellulose content. In contrast, these signals were less evident in the UHEF-ES spectrum, suggesting that the UHEF-pretreated sample retained less residual hemicellulose. The characteristic lignin signals at 55 ppm (methoxyl carbon), 134 ppm (C2/C5 of guaiacyl units), and 152 ppm (C3/C5 of syringyl units) [29] exhibited decreased intensities following both pretreatments, consistent with the partial degradation and removal of lignin. These results are consistent with the compositional analysis and FTIR spectroscopic data.

In addition to compositional analysis, solid-state CP/MAS ^13^C NMR spectroscopy can provide information on cellulose crystallinity at the molecular level. It should be noted that the crystallinity measured by NMR reflects the ordering of cellulose chains within the biomass, which differs from the bulk crystallinity index determined by XRD [30]. As shown in Figure 8, the characteristic signals of crystalline cellulose in the RES spectrum appeared at 64 ppm (C6) and 88 ppm (C4). Following pretreatment, the cellulose signals shifted to 61.5 ppm and 83 ppm, indicating a decrease in crystalline cellulose content [22]. The cellulose crystallinity was calculated from the ratio of the C4 crystalline cellulose signal intensity to the total C4 signal intensity (crystalline + amorphous), and the results are summarized in Table S4. Following UHOF and UHEF pretreatment, the cellulose crystallinity decreased from 55.03% (RES) to 47.51% and 49.07%, respectively, suggesting that both pretreatments disrupted the crystalline domains of cellulose. This reduction in cellulose crystallinity has been shown to improve the hydrolysis efficiency [30].

The apparent discrepancy between XRD and NMR crystallinity data observed in this study has been reported in the literature and can be reconciled by considering the different measurement principles. Zhou et al. noted in their comprehensive review that while XRD measures bulk crystallinity based on diffraction peak intensities, NMR crystallinity reflects the local ordering environment of cellulose chains [3]. The preferential removal of amorphous hemicellulose and lignin can cause an apparent increase in XRD-derived CrI due to the relative enrichment of crystalline cellulose in the residual solid, even as the absolute crystalline domain structure is disrupted, as evidenced by NMR. This dual crystallinity analysis provides a more comprehensive understanding of cellulose structural changes during UHEF pretreatment and highlights the importance of using multiple complementary characterization techniques. Because CP/MAS NMR crystallinity is semi-quantitative, these values are used for relative comparison among samples rather than as absolute crystallinities. The apparent increase in XRD CrI is therefore interpreted as an enrichment of crystalline cellulose in the residue, whereas the NMR C4 analysis indicates the disruption of local cellulose ordering.

### 2.7. SEM Analysis

Changes in the chemical composition of lignocellulose are typically accompanied by alterations in surface morphology, which can influence the accessibility of cellulase, and consequently, the yield of reducing sugars from enzymatic hydrolysis [30]. Therefore, SEM analysis was performed on all ES samples to evaluate the changes in surface microstructure induced by pretreatment.

**Figure 9:**
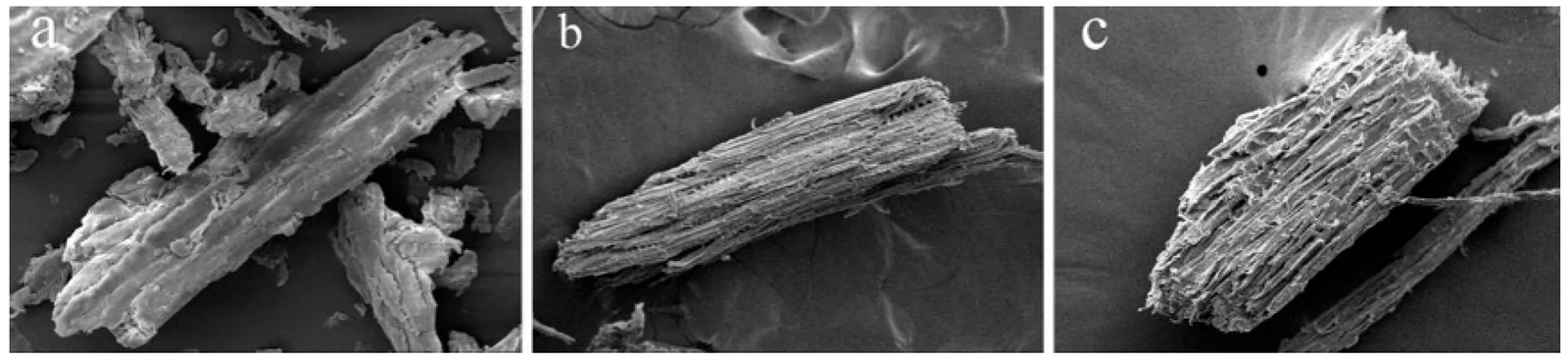
SEM images of RES (**a**), UHOF-ES (**b**), and UHEF-ES (**c**).

As shown in Figure 9a, the surface of raw ES was smooth and compact, exhibiting a rigid and dense structural morphology. This intact surface structure is responsible for the inherent recalcitrance of lignocellulosic biomass to biological degradation [27]. Following pretreatment, numerous grooves, fissures, and cracks were observed on the ES surface. These morphological changes are attributed to the attack of hydroxyl radicals (HO·) on hemicellulose and lignin during pretreatment, resulting in the cleavage and removal of these components (as confirmed by FTIR analysis) and consequent surface roughening [27]. These structural modifications increase the external surface area of ES and provide additional binding sites for cellulase, thereby promoting cellulose hydrolysis and increasing sugar production [30]. Notably, the UHEF-ES surface exhibited a more extensively roughened and porous morphology compared with UHOF-ES, suggesting that UHEF pretreatment would generate a greater quantity of cellulose hydrolysis products.

### 2.8. Enzymatic Hydrolysis and Recycling Analysis

**Figure 10:**
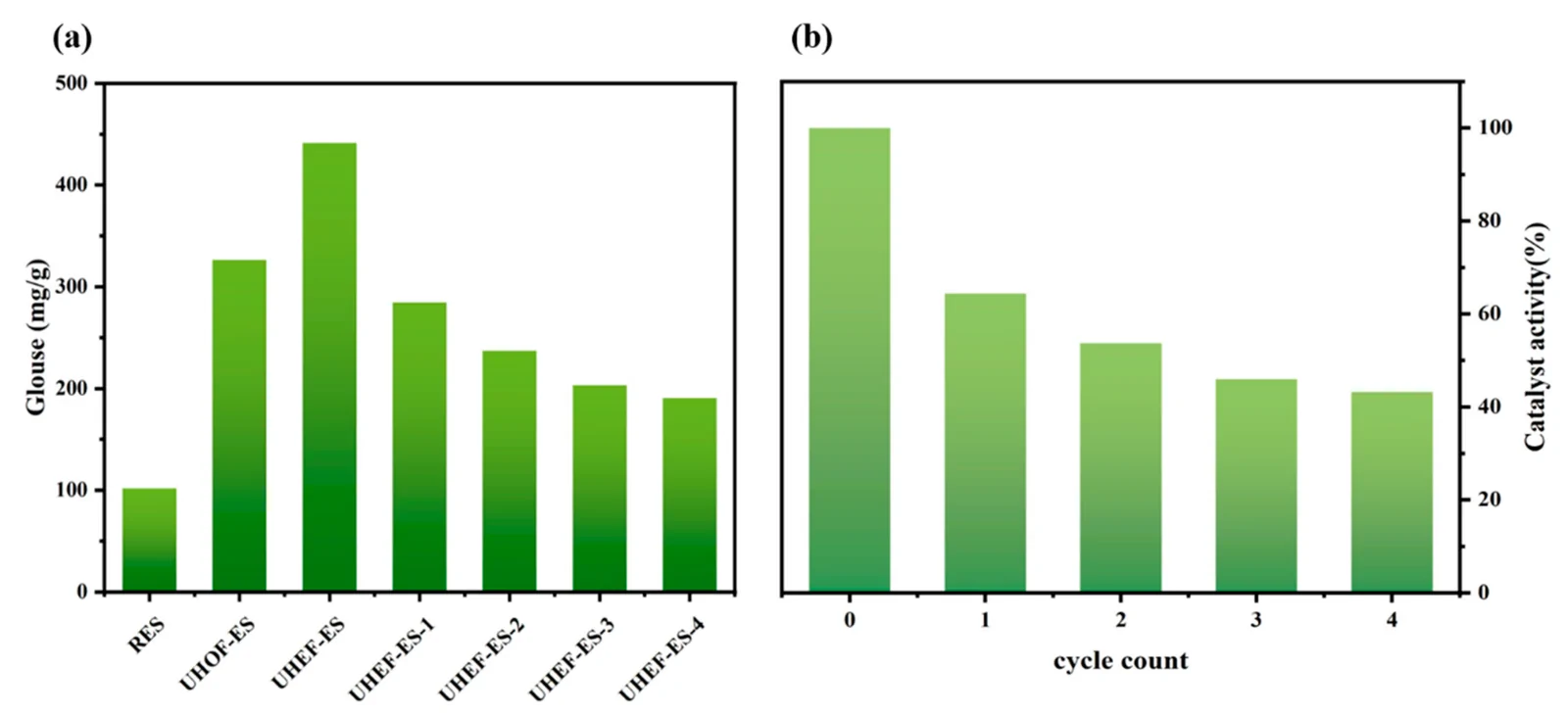
Reducing sugar yield (**a**) and catalyst relative activity (**b**).

The reducing sugar yields from the enzymatic hydrolysis of RES and pretreated ES samples are presented in Figure 10a. The reducing sugar yield of RES was 102.03 ± 4.21 mg/g, which increased significantly following both UHOF and UHEF pretreatments, reaching 326.32 ± 7.92 mg/g and 441.45 ± 6.87 mg/g, respectively. The UHEF-pretreated ES exhibited the highest reducing sugar yield, consistent with our initial expectations. Based on the comprehensive characterization data presented above, this enhanced performance can be attributed to the more extensive removal of lignin and hemicellulose during UHEF pretreatment, which generated numerous surface grooves and increased the external surface area of ES, providing more attachment sites for cellulase. Concurrently, the disruption of cellulose crystalline regions during pretreatment facilitated enhanced cellulase–cellulose interaction, resulting in greater reducing sugar production [21].

To evaluate the recyclability of the iron-loaded zeolite catalyst, recovery and reuse experiments were conducted across multiple pretreatment cycles (Figure 10b). During the first recycling cycle, the reducing sugar yield of ES decreased substantially, falling below that achieved with fresh catalyst. With successive recycling cycles, the reducing sugar yield continued to decline progressively. The relative catalytic activity was calculated based on the reducing sugar yield of UHEF-ES relative to that obtained with fresh catalyst. By the fourth cycle, the catalytic activity had decreased to approximately 43% of its initial value. This progressive deactivation may be attributed to the cavitation and mechanical effects of ultrasonication, which can cause the detachment of Fe^2+^ from the zeolite support, thereby reducing the effective catalyst concentration in the UHEF system and diminishing the pretreatment efficiency [19].

Fe leaching was quantified to clarify the deactivation pathway (Table 3 and Figure S3b). By cycle 4, the cumulative Fe loss reached 20.3 wt% relative to the initial catalyst. This trend is consistent with the progressive oxidation of surface Fe^2+^ and the partial blockage of exchange sites by organic residues. Thus, catalyst deactivation is attributed to the combined effects of Fe leaching, surface oxidation, and fouling rather than to collapse of the zeolite framework.

## 3. Materials and Methods

### 3.1. Materials

Raw eucalyptus sawdust (RES) was collected from the teaching field base of Wood Science at South China Agricultural University (Guangzhou, China). The RES was washed, dried, and screened to a particle size of 60–80 mesh using a vibrating sieve (Model YB-1000A, YUNBANG, Jinhua, China). Hydrogen peroxide solution (H_2_O_2_, 30%, *w*/*w*) and all other chemical reagents were purchased from Shanghai Aladdin Bio-Chem Technology Co., Ltd. (Shanghai, China). Cellulase (Trichoderma viride, E0240) and β-glucosidase (M0031-2) were purchased from Shanghai Bo’ao Biological Technology Co., Ltd. (Shanghai, China). The heterogeneous catalyst was prepared from commercially available natural clinoptilolite (Zhengzhou HengNuo Filter Material Co., Ltd., Zhengzhou, China). The physicochemical characteristics and structural properties of this zeolite are summarized in Table S1.

### 3.2. Methods

#### 3.2.1. Catalyst Preparation and Fe^2+^ Adsorption

The natural zeolite was ground and passed through a 100-mesh sieve, then dried in an oven at 105 °C for 24 h. Subsequently, 5 g of dried zeolite and 100 mL of NaOH solution (8%, *w*/*w*) were placed in a 250 mL conical flask. The flask was irradiated in a microwave oven at 480 W for 5 min. The solid was then separated by centrifugation at 4000 rpm for 10 min and washed with distilled water until the supernatant reached neutral pH. The modified zeolite was subsequently dried at 105 °C for 12 h.

For Fe^2+^ adsorption, 1 g of modified zeolite was added to 100 mL of FeSO_4_ solution at concentrations ranging from 2 to 40 g/L (*w*/*v*). The mixed suspension was stirred at 50 °C for 1 h to maximize Fe^2+^ adsorption onto the zeolite. The Fe^2+^ adsorption efficiency was calculated based on the residual Fe^2+^ concentration in the solution, which was determined by o-phenanthroline complexometry [31]. FeSO_4_·7H_2_O (AR, 99%) was used as the iron source. After adsorption, the solid was separated by centrifugation at 4000 rpm for 10 min, washed with deionized water until the supernatant was neutral, and dried at 60 °C to constant mass before characterization and use.

#### 3.2.2. Pretreatment of RES

The heterogeneous Fenton system was performed under ultrasound irradiation, with ultrasound power and reaction time varied according to the experimental design. The ultrasound-assisted heterogeneous Fenton-like system is denoted UHEF, whereas the corresponding ultrasound-assisted homogeneous Fe^2+^ system is denoted UHOF. A fixed mole ratio of 1:3 of Fe^2+^ to H_2_O_2_, selected from preliminary screening over Fe^2+^:H_2_O_2_ ratios of 1:1–1:5 to maximize •OH generation while limiting H_2_O_2_ scavenging by excess Fe^2+^, was mixed with deionized water and then 1 g of RES was added to the system. Four control experiments were conducted under otherwise identical conditions: ultrasound only, H_2_O_2_ only, homogeneous Fenton without ultrasound (HOF), and heterogeneous Fenton without ultrasound (HEF). After each run, the solid residue was filtered, washed to neutral pH, and dried at 60 °C to constant mass. All pretreatments were performed in triplicate, and results are reported as mean ± SD. Reducing sugar yield was determined by the DNS method at 540 nm using a glucose calibration curve and expressed as mg glucose equivalents per g of dry substrate. The response surface methodology (RSM) with central composite design (CCD) was adopted to obtain optimal pretreatment conditions that displayed the maximum sugar yield. Three factors were used: H_2_O_2_ concentration (*A*), ultrasound power (*B*), and reaction time (*C*). A total of 20 experimental runs, comprising 8 factorial points, 6 axial points (α = 2), and 6 center points were executed. The test condition included an H_2_O_2_ concentration of 2%~6%, reaction times of 50~130 min, and an ultrasound power of 160~320 W. Data analysis was carried out using Design Expert.

#### 3.2.3. Enzymatic Hydrolysis

Enzymatic hydrolysis was performed as follows: 50 mg of pretreated ES was added to a 100 mL flask containing 30 mL of sodium citrate buffer (50 mM, pH 4.8). Cellulase and β-glucosidase were loaded at 35 filter paper units (FPU)/g and 55 cellobiase units (CBU)/g of substrate, respectively. The flask was sealed and incubated in a rotary shaker at 180 rpm and 50 °C for 48 h. Aliquots of the hydrolysate were withdrawn every 4 h, and the reducing sugar concentration was determined by the 3,5-dinitrosalicylic acid (DNS) method [32].

#### 3.2.4. Analysis Methods

The chemical composition of ES before and after pretreatment was determined according to the Van Soest method [33]. Neutral detergent fiber (NDF), acid detergent fiber (ADF), and acid detergent lignin (ADL) were sequentially prepared by extracting samples with neutral detergent, acid detergent, and 72% (*v*/*v*) H_2_SO_4_, respectively. The contents of hemicellulose, cellulose, and Klason lignin were calculated as the differences between NDF and ADF, ADF and ADL, and ADL and ash, respectively. The relative percentage difference between replicate measurements was maintained below 5% to ensure reproducibility.

For FTIR spectroscopic analysis, ES samples were ground in a Wiley mill to a particle size of 0.2 mm (IKA MF10, IKA-Werke, Staufen, Germany). The samples were thoroughly mixed with spectroscopic-grade KBr and pressed into translucent pellets. The spectra were recorded in the range of 4000–400 cm^−1^ at a spectral resolution of 4 cm^−1^ using a NEXUS 670 FTIR spectrometer (Thermo Nicolet Corporation, Madison, WI, USA).

X-ray powder diffraction (XRD) analysis was performed using an Empyrean diffractometer (PANalytical B.V., Almelo, The Netherlands) over a 2θ range of 5° to 50° at a scan step size of 0.03°. The crystallinity index (CrI) was calculated using the Segal method (Equation (2)) [23]:

(2) $$\mathrm{CrI}=(I_{002}-I_{\mathrm{am}})/I_{002}\times 100\%$$

where I_002_ is the intensity of the peak at a 2θ value of 22.5°, and I_am_ is the intensity at a 2θ value of 19°.

Solid-state ^13^C CP/MAS NMR spectroscopy was performed using a Bruker (Billerica, MA, USA) Avance 400 MHz NMR spectrometer equipped with a 4 mm CP/MAS solid-state probe. The spectra were acquired at a magic-angle spinning (MAS) frequency of 15 kHz. Cross-polarization was performed with a contact time of 2 ms, a recycle delay of 4 s, and 2048 accumulated scans; because CP/MAS is not strictly quantitative, the NMR crystallinity values are interpreted as semi-quantitative. Cellulose crystallinity was estimated by fitting the C4 region (80–92 ppm) with Gaussian–Lorentzian functions, assigning 86–92 ppm to crystalline C4 and 80–86 ppm to amorphous C4. The catalyst morphology and elemental composition were characterized by scanning electron microscopy coupled with energy-dispersive X-ray spectroscopy (SEM-EDX, S-570, ZEISS, Oberkochen, Germany). X-ray photoelectron spectroscopy (XPS) was performed on a Thermo Scientific K-Alpha spectrometer (Al Kα, 1486.6 eV), with charge correction to adventitious C 1s at 284.8 eV. Elemental composition was determined by ICP-OES (Agilent 5110, Santa Clara, CA, USA) after microwave-assisted acid digestion. Fe leaching after each reuse cycle was quantified by ICP-OES of the filtrate, and cumulative Fe loss was calculated relative to the initial Fe content of the catalyst.

## 4. Conclusions

In this study, an ultrasound-assisted heterogeneous Fenton-like (UHEF) system was constructed by combining Fe^2+^-loaded clinoptilolite with H_2_O_2_ for eucalyptus sawdust pretreatment. XRD, XPS, and ICP-OES results confirmed that iron was immobilized on the zeolite mainly as exchanged Fe^2+^ with minor surface Fe^3+^/FeOOH species, giving a total Fe content of 16.92 wt%. Control experiments showed that ultrasound or H_2_O_2_ alone had limited effects, whereas the combination of ultrasound with the heterogeneous Fenton-like system produced the highest reducing sugar yield (441.45 ± 6.87 mg/g), 4.32-fold that of RES. The enhancement is associated with the partial removal of lignin and hemicellulose, the disruption of cellulose ordering, and increased surface accessibility. Recycling tests showed that the catalyst retained partial activity over four cycles, but Fe leaching (cumulative loss of 20.3 wt%) and probable surface oxidation/fouling limit its long-term stability. Future work should focus on improving Fe anchoring and regenerating the catalyst before practical biorefinery application.

## Figures and Tables

**Table 1:** ICP-OES elemental and oxide composition of the zeolite catalysts.

Sample	SiO_2_	Al_2_O_3_	Fe_2_O_3_	Na_2_O	K_2_O	CaO	MgO	Fe	S
NaOH-modified clinoptilolite	67.50	12.35	1.23	4.61	1.14	0.87	0.63	0.86	0.02
Fe^2+^-loaded clinoptilolite	56.57	10.36	24.19	3.86	0.96	0.73	0.53	16.92	0.48

**Table 2:** Analysis of variance (ANOVA) for the experimental results of the CCD.

Source	Sum of Squares	df	Mean Square	F-Value	*p*-Value Prob > F
Model	22,367.07	9	2485.23	19.90	<0.0001
A	1777.04	1	1777.04	14.23	0.0036
B	383.57	1	383.57	3.07	0.1102
C	9526.74	1	9526.74	76.28	<0.0001
AB	1791.61	1	1791.61	14.35	0.0036
AC	295.00	1	295.00	2.36	0.1553
BC	587.22	1	587.22	4.70	0.0553
A^2^	711.88	1	711.88	5.70	0.0381
B^2^	2561.53	1	2561.53	20.51	0.0011
C^2^	6863.64	1	6863.64	54.96	<0.0001
Residual	1248.89	10	124.89		
Lack of Fit	662.42	5	132.48	1.13	0.4485
Pure Error	586.47	5	117.29		
Cor Total	23,615.96	19			
R^2^ = 0.9471, Adjusted R^2^ = 0.8995					

**Table 3:** Fe leaching and cumulative Fe loss during catalyst reuse.

Cycle	Leached Fe (mg/L)	Incremental Fe Loss (wt%)	Cumulative Fe Loss (wt%)
1	42.6	6.80	6.80
2	35.1	5.60	12.40
3	27.8	4.44	16.84
4	21.7	3.46	20.30

## Data Availability

The original contributions presented in this study are included in the article/Supplementary Materials. Further inquiries can be directed to the corresponding author.

## Supplementary Materials

The following supporting information can be downloaded at: https://www.mdpi.com/article/10.3390/molecules31162926/s1, Figure S1. Adsorption percentage for ferrous ions coated on zeolite (the mass ratio of zeolite to ferrous sulfate was set as 1/0.2, 1/0.5, 1/0.8, 1/1, 1/2, 1/3, 1/4, respectively). Figure S2. Response surface curves for sugar yield showing the interaction between (a) concentration of H_2_O_2_ and pretreatment time at B = 0, (b) ultrasound power and concentration of H2O2 at C = 0, and (c) ultrasound power and pretreatment time at A = 0. Figure S3. Control experiments and Fe leaching during catalyst reuse: (a) reducing sugar yields of control and Fenton pretreatments (mean ± SD, n = 3); (b) leached Fe concentration and cumulative Fe loss over four reuse cycles. Table S1. Characteristics of the clinoptilolite used in this study (provided by producer). Table S2. Coded values for each variable of the CCD for sugar yield. Table S3. Reducing sugar yields of the control and Fenton pretreatment experiments (mean ± SD, n = 3). Table S4. Crystallization index of ES before and after pretreatment.
