# Supplementary material for: Ultrasonic-Assisted Heterogeneous Fenton-like (UHEF) Pretreatment of Eucalyptus Sawdust for Enhanced Enzymatic Hydrolysis
Source: Molecules. 2026 Aug 21;31(16):2926. doi: 10.3390/molecules31162926 (PMC13516775; doi:10.3390/molecules31162926)
Supplement: Supplementary file 1 [file molecules-31-02926-s001.zip › molecules-4452961-supplementary.pdf]

Supporting Information

Ultrasonic-Assisted Heterogeneous Fenton-like (UHEF) Pretreatment of Eucalyptus Sawdust for Enhanced Enzymatic Hydrolysis

Han Zhang, Cuixian Peng, Xiaoguo Wang, Ping Li, Wei Tan and Shujie Wang*

Contents: Figure S1; Figure S2; Figure S3; Table S1; Table S2; Table S3; Table S4.

Table S1. Characteristics of the clinoptilolite used in this study (provided by producer).

Zeolite	Size (mm)	Surface area (m^2/g)	SiO_2 (wt.%)	Al_2O_3 (wt.%)
Natural clinoptilolite	0.15~0.18	700	67.50	12.35

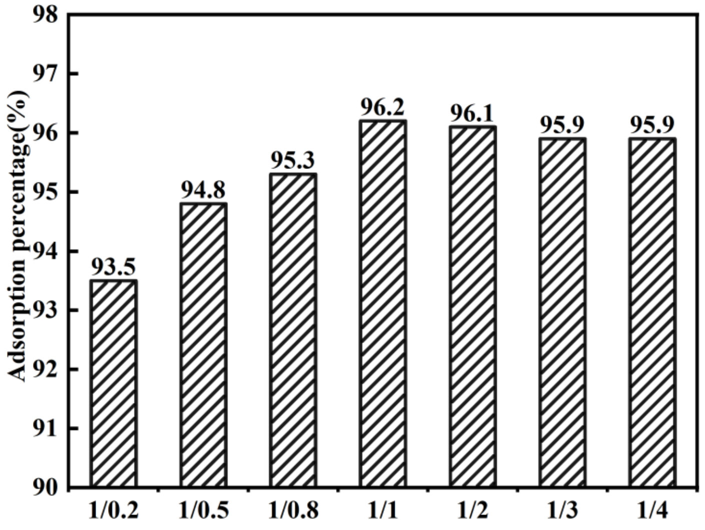

Figure S1. Adsorption percentage for ferrous ion coated on zeolite (the mass ratio of zeolite to ferrous sulfate was set as 1/0.2, 1/0.5, 1/0.8, 1/1, 1/2, 1/3, 1/4, respectively).

Table S2. Coded values for each variable of the CCD for sugar yield.

Variables	Unit	-α	-1	0	+1	+α
A:H_2O_2 concentration	%	2	3	4	5	6
B:Ultrasound power	W	160	200	240	280	320
C:Pretreatment time	min	50	70	90	110	130

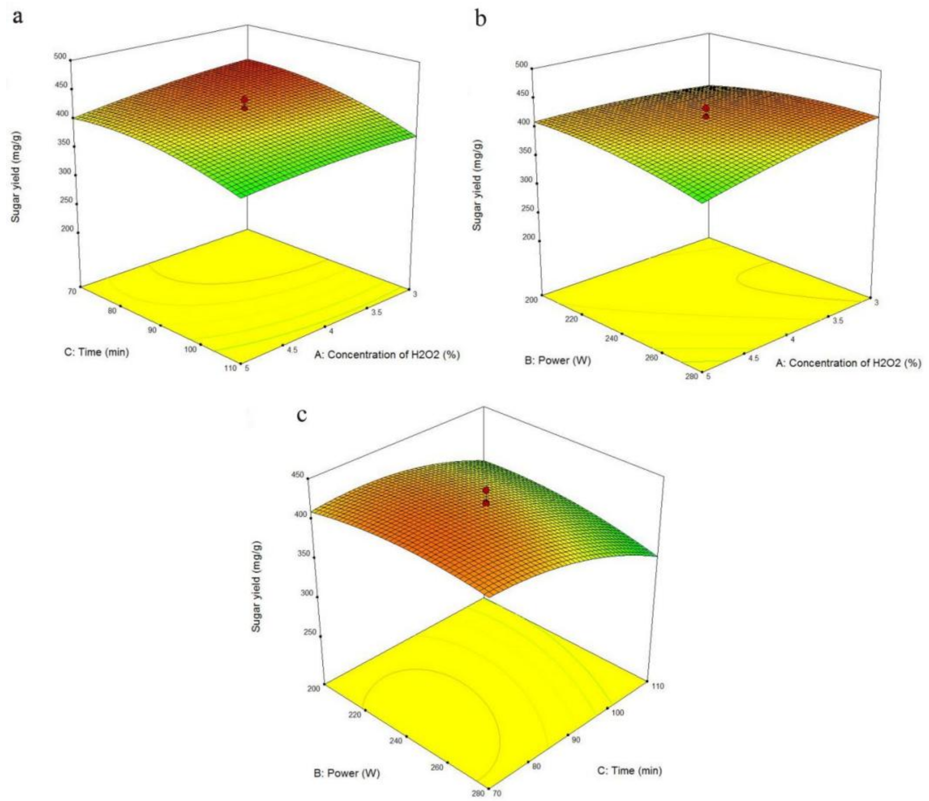

Figure S2. Response surface curves for sugar yield showing the interaction between (a) concentration of H_2O_2 and pretreatment time at B = 0, (b) ultrasound power and concentration of H_2O_2 at C = 0, and (c) ultrasound power and pretreatment time at A = 0.

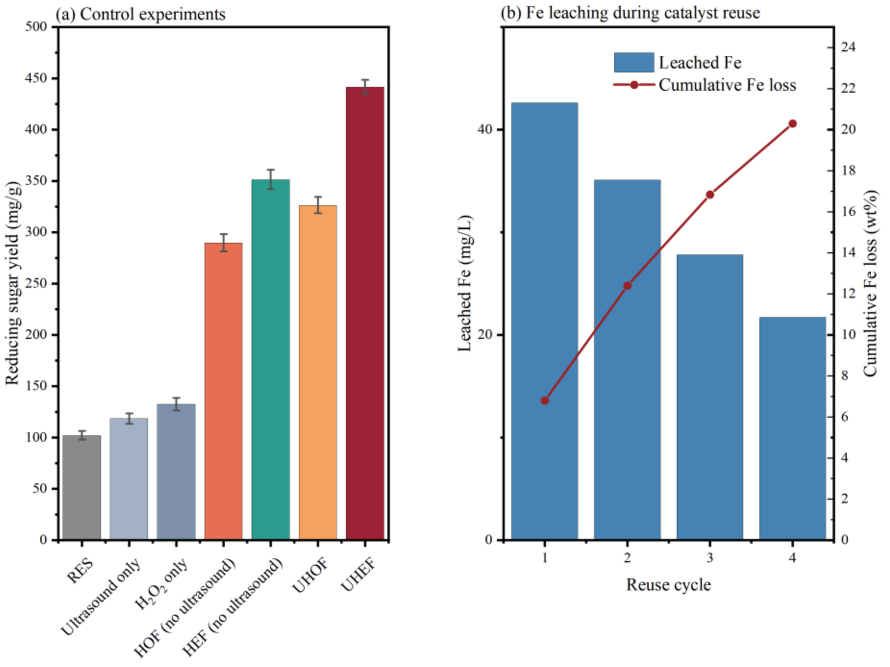

Figure S3. Control experiments and Fe leaching during catalyst reuse: (a) reducing sugar yields of control and Fenton pretreatments (mean ± SD, n = 3); (b) leached Fe concentration and cumulative Fe loss over four reuse cycles.

Table S3. Reducing sugar yields of control and Fenton pretreatment experiments (mean ± SD, n = 3).

Experiment	Reducing sugar yield (mg/g)
RES	102.03 ± 4.21
Ultrasound only	118.35 ± 5.02
H2O2 only	132.48 ± 6.15
HOF (no ultrasound)	289.64 ± 8.33
HEF (no ultrasound)	351.27 ± 9.48
UHOF	326.32 ± 7.92
UHEF	441.45 ± 6.87

Table S4. Crystallization index of ES before and after pretreatment.

Sample	RES	UHOF-ES	UHEF-ES
CrI for substrate (%)	53.80	52.67	55.98
CrI for cellulose (%)	55.03	47.51	49.07
